# Identifying Hemostatic Thresholds in Cancer Patients Undergoing Coronary Angiography Based on Platelet Count and Thromboelastography

**DOI:** 10.3389/fcvm.2020.00009

**Published:** 2020-02-14

**Authors:** Ali M. Agha, Clarence Gill, Dinu Valentin Balanescu, Teodora Donisan, Nicolas Palaskas, Juan Lopez-Mattei, Saamir Hassan, Peter Y. Kim, Konstantinos Charitakis, Mehmet Cilingiroglu, Thein Hlaing Oo, Michael Kroll, Jean Bernard Durand, Cheryl Hirsch-Ginsberg, Konstantinos Marmagkiolis, Cezar Iliescu

**Affiliations:** ^1^Department of Internal Medicine, University of Texas Health Science Center at Houston, Houston, TX, United States; ^2^Department of Cardiology, University of Texas Health Science Center at Houston, Houston, TX, United States; ^3^Department of Cardiology, MD Anderson Cancer Center, Houston, TX, United States; ^4^Department of Cardiology, University of Arkansas, Little Rock, AR, United States; ^5^Department of Hematology, MD Anderson Cancer Center, Houston, TX, United States; ^6^Department of Pathology, MD Anderson Cancer Center, Houston, TX, United States; ^7^Department of Cardiology, Advent Health, Zephyrhills, FL, United States

**Keywords:** thromboelastogarphy, cancer, thrombocytopenia, coronary angiography, mortality

## Abstract

**Objectives:** To evaluate the role of platelet count and thromboelastogram (TEG) in the treatment of thrombocytopenic cancer patients with suspected coronary artery disease (CAD).

**Background:** Cancer patients with CAD and thrombocytopenia are often treated non-invasively (i.e., without coronary angiography when clinically indicated) due to perceived high risk of bleeding. We sought to evaluate coagulability based on TEG and determine if platelet count and TEG could predict bleeding risk/mortality among cancer patients undergoing coronary angiography (CA).

**Methods:** Baseline demographics, platelet count, and TEG parameters were recorded among cancer patients that underwent CA and had a concomitant TEG. Logistic regression and univariate proportional hazards regression analysis were performed to determine the impact of platelet count and coagulability on 24-month overall survival (OS).

**Results:** All patients with platelet count <20,000/mm^3^ and nearly all patients with platelet count 20,000–49,000/mm^3^ were hypocoagulable based on TEG results. In contrast, nearly all patients with platelet counts of 50,000–99,999/mm^3^ had normal TEG results and OS similar to those with platelet counts of ≥100,000/mm^3^. Coagulability based on TEG was not associated with OS. However, a platelet count of <50,000/mm^3^ was associated with worse 24-month OS (hazard ratio = 2.76; *p* = 0.0072) when compared with a platelet count of ≥100,000/mm^3^. No major bleeding complications were observed in all groups.

**Conclusion:** The majority of cancer patients with platelet counts of <50,000/mm^3^ were hypocoagulable based on TEG and had worse OS at 24 months. The relatively normal TEGs in the >50,000/mm^3^ groups, as well as the improved survival, suggest that with appropriate clinical indication and risk/benefit assessment, a cut-off of 50,000/mm^3^ platelets can be considered for CA in cancer patients.

## Introduction

With the development and advancement of modern cancer therapies, the survival of patients with cancer has substantially improved. Aging is associated with increased risk of both coronary artery disease (CAD) and cancer. Cancer therapies (i.e., mediastinal radiation and chemotherapy) can accelerate vascular aging (1, 2). Cardiologists are increasingly involved in the care of cancer patients, with the intention of improving their overall survival (OS) (3) and quality of life—this is reflected by the increased number of cardiac interventions now being performed among cancer patients (4).

Chronic thrombocytopenia is a frequently recognized complication of cancer and its treatment, and is considered a relative contraindication for coronary angiography (CA). Approximately 10% of cancer patients have a platelet count of <100,000/mm^3^ (5). Causes of chronic thrombocytopenia in cancer patients are multifactorial, but frequently include systemic chemotherapy (6). Although thrombocytopenia is traditionally defined as platelet count <150,000/mm^3^, concern for CA increases as thrombocytopenia increases in severity: mild thrombocytopenia is defined as a platelet count between 50,000–99,999/mm^3^, moderate thrombocytopenia is defined as a platelet count between 20–49,000/mm^3^ and severe thrombocytopenia is defined as a platelet count <20,000 mm^3^ (7). Thrombocytopenia has been associated with an increased risk of both ischemic and bleeding complications during CA (8–10). However, recent experiences with coronary interventions in patients with thrombocytopenia showed encouraging results with respect to safety, suggesting that platelet function could trump absolute platelet counts when assessing procedure-related bleeding risk (6).

Thromboelastography (TEG) is a hemostatic test that measures the clotting efficacy of whole blood by emulating an environment similar to the venous flow in the body. TEG can provide dynamic measures of the kinetics, strength, and stability of fibrin clotting, which is influenced by both platelet function and platelet-fibrinogen interaction (11, 12) and has been studied extensively in cardiovascular surgery literature (13). Bleeding complications have a direct impact on survivorship among cancer patients (14). Hemostasis appears to be affected more than platelet adhesion in thrombocytopenic patients (15). Despite a substantial decrease in bleeding rates during CA (16) as a result of more frequent use of radial approach (17) and more meticulous femoral access techniques utilizing micropuncture, vascular Doppler ultrasound (18) and vascular closure devices, concern for bleeding complications in cancer patients with suspected CAD and thrombocytopenia still remains. As a consequence, many of these patients are treated non-invasively (19). TEG provides real-time hemostatic parameters, and its results could influence the selection of blood products required.

This study sought to determine whether TEG results can identify and modify hemostatic thresholds, thereby providing a more comprehensive risk stratification before proceeding with CA, in addition to helping tailor the appropriate blood product administration when bleeding complications do occur.

## Methods

All cancer patients with suspected CAD and included in The University of Texas MD Anderson Cancer Center cardiac catheterization laboratory registry that underwent pre-procedural TEG between January 1st, 2009, and December 31st, 2017, were included. Baseline demographics, medical, family, procedural, and social history (6), traditional cardiovascular risk factors (hypertension, hyperlipidemia, family history, smoking history, and diabetes), and laboratory data including complete blood count, coagulation parameters (international normalized ratio, prothrombin time, and partial thromboplastin time), serum creatinine level, fasting lipid panel, glycosylated hemoglobin level, and fasting plasma glucose level were included. The MD Anderson Institutional Review Board approved this study and waived the need for informed consent owing to its retrospective nature.

Platelet count on the day of CA was used to classify patients into 4 groups: normal ≥100,000/mm^3^, mild thrombocytopenia 50,000–99,999/mm^3^, moderate thrombocytopenia 20–49,999/mm^3^, and severe thrombocytopenia <20,000/mm^3^.

Arterial access was obtained using a modified Seldinger technique with a micropuncture kit. The decision for the access site was based on modified Allen or Barbeau test findings and operator preference (6, 20), although radial was preferred over femoral. Vascular Doppler ultrasound was used for most cases of radial access and in femoral access, depending on operator preference.

Whole blood was analyzed for its hemorrhagic and thrombotic potential using a TEG 5000 Thrombelastography Hemostasis Analyzer (Haemonetics, Braintree, Massachusetts) according to the standard procedures outlined in its user guide. The standard TEG test was performed once using the patient's whole blood only (producing results of the “S1 channel”) and once using the patient's whole blood plus heparinase (producing results of the “heparinase channel”), and the values representing clot formation determined by this test were recorded. The parameters (values) measured by the TEG are based on a graphical representation of the ability of a developing clot to couple the movement of an oscillating cup with a suspended torsion wire pin. The graph normally progresses from a phase of no movement (baseline) at the onset of the analysis through the development of a stable clot and follows that clot for 30 min to record clot dissolution. The main values of interest in this study include: the reaction time (R, measured in minutes), which represents the time from test initiation until a small clot (2 mm of deviation from baseline) is formed; the K (measured in minutes), which is the time for the deviation from baseline to move from 2 mm (R) to 20 mm and represents the speed of clot formation; the alpha angle (a) is another measure of clot kinetics, which is the tangent of the curve from the first detectable deviation from baseline until the K value is reached (or MA is reached in instances when the clot never reaches 20 mm); and the maximum amplitude (MA, measured in millimeters) of the deviation from baseline, which represents clot strength; the G value (dynes/cm^2^), which is a log derivation of the MA and also represents the clot strength; clot lysis (LY30), which is based on the percent reduction of the area under the curve from the time the MA is measured until 30 min after that time point and finally, the coagulation index (CI, reference ranges −3 to +3) which is a value derived from the R, K, MA, and alpha angle (a) that is meant to describe the patient's overall coagulation profile. The TEG results are reviewed and interpreted by a board-certified hematopathologist to determine the coagulability of each whole blood sample. Hypocoagulability on TEG at our institution is defined as a prolonged R time, a low alpha angle, or a low MA.

Bleeding was defined according to the Bleeding Academic Research Consortium bleeding criteria in patients with acute coronary syndrome (21), where a major bleeding complication was considered to be a BARC 3 or greater bleeding event. This was identified by searching the electronic medical records for pre-specified drop in hemoglobin and key words such as “bleeding,” “hematoma,” “hemorrhage,” “pseudoaneurysm,” and “transfusion,” as described previously (6), and by scheduled surveillance telephone contact and/or outpatient visits with family and external providers. Any bleeding complications up to the day of last follow-up or date of death were recorded.

Demographic characteristics for the study cohort as a whole and by platelet count at the time of TEG, as well as by coagulability on TEG (hypocoagulable, hyper-coagulable, or normal), were summarized using mean and standard deviation (SD) and/or using median and range for continuous variables and using counts and percentages for categorical variables. Platelet groups and coagulability groups were compared by analysis of variance or Kruskal-Wallis test for continuous variables and by chi-square test or Fisher exact test for categorical variables. Logistic regression analyses were performed to identify variables associated with hypocoagulability. OS was defined as the time from the CA to death or last follow-up. OS was censored at 24 months, and survival status at 24 months was obtained. The Kaplan-Meier method and log-rank test were used to compare OS between subgroups. Univariate Cox proportional hazards regression analyses were conducted to identify variables associated with OS. A *p*-value of <0.05 indicated statistical significance. SAS 9.4 (SAS Institute Inc.) was used for all data analysis.

## Results

Demographic characteristics and laboratory values are summarized in Tables 1, 2. A total of 70 patients diagnosed with cancer who underwent cardiac catheterizations with pre-procedural TEGs were included and were followed for a maximum of 24 months after cardiac catheterization. The patients were predominantly men (70.0%), with a mean age of 66.8 ± 11.1 years. There was a high prevalence of traditional cardiovascular risk factors: over one-third of patients had diabetes (34.3%), more than three-fourths had hypertension (78.6%), and more than three-fourths had dyslipidemia (78.6%). Hematologic malignancies were more prevalent (44 cases, 62.9%) than solid malignancies (26 cases, 37.1%).

**Table 1:** Cohort demographic and metabolic characteristics, continuous variables (*n* = 70).

**Continuous variable**	**Mean ± SD**	**Median**	**Range**
Age (y)	66.77 ± 11.05	68	41, 91
Weight (kg)	84.87 ± 22.29	83.5	34.7, 145
Body surface area (m^2^)	1.98 ± 0.27	1.99	1.53, 2.58
Baseline platelet count (K/mm^3^)	102.3 ± 107.91	61.5	4, 489
Absolute neutrophil count (K/mm^3^)	6.13 ± 10.02	3.16	0, 48.7
International normalized ratio	1.6 ± 3.07	1.18	0.97, 26.9
Creatinine (mg/dL)	1.23 ± 0.98	0.99	0.48, 5.92
Hemoglobin (g/dL)	10.06 ± 1.36	9.9	8, 14.6
Platelet count on day of TEG (K/mm^3^)	104.92 ± 106.33	64	3, 489
Prothrombin time (s)	15.66 ± 1.96	15.2	13, 21.8
Partial thromboplastin time (s)	34.15 ± 7.913	33.5	1.05, 52.9
S1 channel			
TEG R value	6.68 ± 7.67	4.9	2.6, 64.2
TEG K value	2.64 ± 3.08	1.7	0.8, 19.8
TEG angle	62.46 ± 13.95	65.95	15.1, 80.4
TEG MA value	56.38 ± 15.22	59.1	18.8, 83.8
TEG G value	7.99 ± 5.11	7.25	0, 25.9
TEG EPL value	0.53 ± 1.73	0	0, 10.4
TEG Ci value	−0.6 ± 4.49	0.2	−23, 5.9
Heparinase channel			
TEG R value	5.38 ± 1.94	4.9	2.6, 11.8
TEG K value	2.33 ± 2.03	1.75	0.8, 14.9
TEG angle	63.49 ± 13.45	66.8	16.8, 80.6
TEG MA value	55.23 ± 14.79	55.9	18.3, 80.4
TEG G value	7.45 ± 4.47	6.3	0, 20.6
TEG EPL value	0.49 ± 1.08	0	0, 6.4
TEG Ci value	−0.31 ± 3.65	0.15	−16.5, 5.2

*SD, standard deviation; TEG, thromboelastography; R value, time to clot detection; K value, speed of clot formation; MA, maximum amplitude; G value, clot strength; EPL, estimated percent lysis; Ci, coagulation index for overall coagulability*.

**Table 2:** Cohort demographic and metabolic characteristics, categorical variables (*n* = 70).

**Categorical variable**	**No. (%)**
Sex	
Male	49 (70.0)
Female	21 (30.0)
Race/ethnicity	
White	47 (67.1)
Black	13 (18.6)
Other	10 (14.3)
Indication	
Acute coronary syndrome	27 (38.6)
Unstable angina	17 (24.3)
Other	26 (37.1)
Cancer type	
Hematologic	44 (62.9)
Solid	26 (37.1)
Diagnosis of hypertension	55 (78.6)
Diagnosis of dyslipidemia	55 (78.6)
Prior congestive heart failure	23 (32.9)
Prior myocardial infarction	23 (32.9)
Prior cerebrovascular disease	3 (4.3)
Diagnosis of diabetes mellitus	24 (34.3)
**Major bleeding event**	**0 (0)**
Aspirin use	32 (45.7)
Statin use	46 (65.7)
Status at last follow-up	
Alive	28 (40)
Dead, cancer-related	30 (42.9)
Dead, other cause	12 (17.1)
Platelet count	
≥100,000 mm^3^	24 (34.8)
50,000–99,999 mm^3^	19 (27.5)
<50,000 mm^3^	26 (37.7)
Coagulation profile	
Normal	24 (34.8)
Hypercoagulable	16 (23.2)
Hypocoagulable	29 (42.0)

*Bold values imply statistical significance (p < 0.05)*.

Mild thrombocytopenia (platelet count 50,000–99,999/mm^3^) was found in 19 (27.1) patients, moderate thrombocytopenia (platelet count 20,000–49,999/mm^3^) was found in 12 (17.1%) patients, and severe thrombocytopenia (platelet count <20,000/mm^3^) was found in 14 (20%) patients.

TEG review by the hemato-pathologist revealed that 29 (42.0%) patients had a hypocoagulable profile. A platelet count of <50,000/mm^3^ was significantly associated with an increased risk of hypocoagulability on TEG (*p* < 0.0001) compared with platelet count ≥100,000/mm^3^. All patients with severe thrombocytopenia were hypocoagulable on TEG, whereas 75% of patients with moderate thrombocytopenia were hypocoagulable on TEG. On the other hand, only 21.1% of patients with mild thrombocytopenia were hypocoagulable on TEG, and only 8.3% of patients with platelet count ≥100,000/mm^3^ were hypocoagulable on TEG (Table 3).

**Table 3:** Percentage of patients with abnormal TEG parameter, by platelet group.

**Platelet group**	**Elevated R (%)**	**Low alpha angle (%)**	**Low MA (%)**	**% of patients with hypocoagulable TEG (based on pathologist review)**
Severe thrombocytopenia (platelet count <20,000 mm^3^)	28.6	50	92.9	100
Moderate thrombocytopenia (platelet count 20,000–49,999 mm^3^)	0	16.7	66.7	75.0
Mild thrombocytopenia (platelet count 50,000–99,999 mm^3^)	15.8	32	21	21.1
Platelet count >100,000 mm^3^	0	0	14.3	8.33

In the severe thrombocytopenia group, a prolonged R time (delay in the time until an initial fibrin clot is detected) was noted in 28.6% of patients, a low alpha angle (which reflects a slow rate of fibrin clot formation) in 50% of patients and a low MA (which reflects a weak strength of the fibrin clot) in 92.9% of patients (Table 3). Among those with severe thrombocytopenia, all 14 (100%) were hypocoagulable on TEG. Likewise, a vast majority of those with moderate thrombocytopenia (9 of 12, 75%) were hypocoagulable on TEG.

Lower baseline platelet count and baseline hemoglobin level (as continuous variables) were also associated with hypocoagulability (*p* < 0.0001 and *p* = 0.0485, respectively), however, a platelet count of 50,000–99,999/mm^3^ was not associated with a hypocoagulable state (*p* = 0.2465) when compared with platelet count ≥100,000/mm^3^.

A hypocoagulable state was not associated with worse OS when compared to normal coagulability [hazard ratio (HR) = 1.771, 95% confidence interval (CI) 0.86–3.63; *p* = 0.1188]

**Figure 1:**
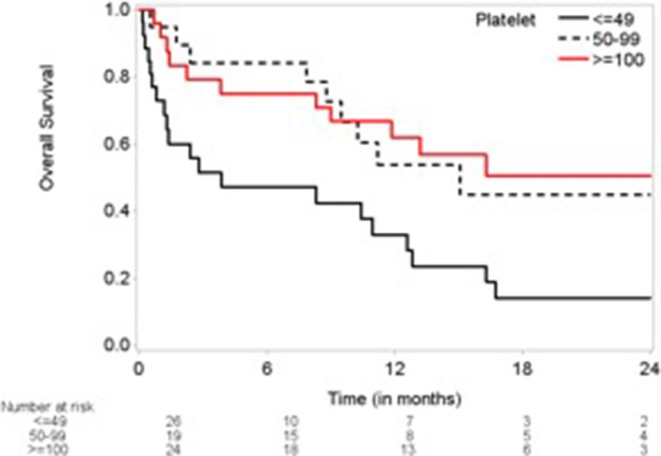
Kaplan-Meier plot of overall survival by platelet level.

On univariate analysis of OS, a platelet count of <50,000/mm^3^ on day of procedure was associated with worse OS at 24 months (HR = 2.757, 95% CI 1.316–5.775; *p* = 0.0072) compared with >100,000/mm^3^ (Figure 1). Other variables associated with worse OS were a higher PT (HR = 1.173, 95% CI 1.027–1.340), prior history of congestive heart failure (HR = 1.92, 95% CI 1.02–3.61; *p* = 0.042), and prior cerebrovascular disease (HR = 5.43, 95% CI 1.16–25.4; *p* = 0.032). Associated with improved OS were a higher hemoglobin level (HR = 0.55, 95% CI 0.41–0.75; *p* = 0.0002), aspirin use (HR = 0.48, 95% CI 0.25–0.91; *p* = 0.025), and statin use (HR = 0.44, 95% CI 0.24–0.82; *p* = 0.0102) (Tables 4, 5). There was no association between hypocoagulability based on TEG and prothrombin time, partial thromboplastin time, or international normalized ratio.

**Table 4:** Prediction of overall survival at 24 months by continuous variables (univariate cox regression).

**Continuous variable**	**Hazard ratio**	**95% CI**	***P*-value**
Age	1.009	0.980–1.038	0.5469
Weight	0.990	0.977–1.004	0.1750
Body surface area	0.218	0.046–1.030	0.0545
Baseline platelet count	0.995	0.991–1.000	**0.0431**
Absolute neutrophil count	1.041	1.014–1.070	**0.0033**
Prothrombin time	1.173	1.027–1.340	**0.0190**
Partial thromboplastin time	1.005	0.958–1.054	0.8470
International normalized ratio	1.052	0.977–1.133	0.1806
Creatinine	0.971	0.721–1.308	0.8480
Hemoglobin	0.554	0.408–0.753	**0.0002**
Platelet count on day of TEG	0.997	0.993–1.000	0.0833
S1 channel			
TEG R value	1.010	0.974–1.048	0.5811
TEG K value	1.048	0.940–1.168	0.3997
TEG angle	0.996	0.971–1.021	0.7376
TEG MA value	0.980	0.958–1.002	0.0757
TEG G value	0.960	0.892–1.033	0.2741
TEG EPL value	0.817	0.580–1.153	0.2507
TEG Ci value	0.972	0.901–1.048	0.4554
Heparinase channel			
TEG R value	1.057	0.901–1.239	0.4967
TEG K value	1.133	0.986–1.301	0.0773
TEG angle	0.986	0.963–1.010	0.2394
TEG MA value	0.980	0.958–1.002	0.0737
TEG G value	0.944	0.872–1.022	0.1555
TEG EPL value	0.845	0.596–1.198	0.3438
TEG Ci value	0.926	0.849–1.011	0.0855

*CI, confidence interval; TEG, thromboelastography; R value, time to clot detection; K value, speed of clot formation; MA, maximum amplitude; G value, clot strength; EPL, estimated percent lysis; Ci, coagulation index for overall coagulability. Bold values imply statistical significance (p < 0.05)*.

**Table 5:** Prediction of overall survival at 24 months by categorical variables (univariate cox regression).

**Categorical variable**	**Hazard ratio**	**95% CI**	**P value**
Sex			
Male	1.000		
Female	0.726	0.377–1.398	0.3379
Race/ethnicity			
White	1.000		
Black	0.505	0.155–1.646	0.2573
Other	0.991	0.465–2.111	0.9817
Indication			
Acute coronary syndrome	1.000		
Unstable angina	1.149	0.574–2.302	0.6946
Other	0.702	0.300–1.642	0.4147
Cancer type			
Hematologic	1.000		
Solid	0.664	0.338–1.308	0.2369
Diagnosis of hypertension	1.038	0.493–2.184	0.9222
Diagnosis of dyslipidemia	0.543	0.270–1.091	0.0862
Prior myocardial infarction	1.214	0.634–2.326	0.5588
Prior congestive heart failure	1.921	1.023–3.608	**0.0422**
Prior cerebrovascular disease	5.428	1.158–25.446	**0.0319**
Diagnosis of diabetes mellitus	0.985	0.508–1.910	0.9651
Aspirin use	0.475	0.247–0.911	**0.0250**
Statin use	0.440	0.235–0.823	**0.0102**
Platelet count			
≥100,000 mm^3^	1.000		
50,000–99,999 mm^3^	1.098	0.454–2.652	0.8361
<50,000 mm^3^	2.757	1.316–5.775	**0.0072**
Coagulation profile			
Normal coagulation	1.000		
Hypocoagulation	0.565	0.275–1.158	0.1188
Hypercoagulable	0.594	0.261–1.350	0.2135

*CI, confidence interval. Bold values imply statistical significance (p < 0.05)*.

Multivariate analysis, which included platelet count and prothrombin time (PT), revealed that a platelet count of <50,000/mm^3^ at TEG time was associated with worse OS at 24 months (HR = 2.392, 95% CI 1.129–5.069; *p* = 0.0229) (Table 6).

**Table 6:** Multivariate analysis: prediction of overall survival at 24 months.

**Variable**	**Hazard ratio**	**95% CI**	***P*-value**
Platelet count <50,000 mm^3^	2.392	1.129–5.069	**0.0229**
Platelet count 50,000–99,999 mm^3^	0.942	0.381–2.328	0.8967
Platelet count ≥100,000 mm^3^	1.000		
Prothrombin Time (PT)	1.151	0.987–1.342	0.0720

*Bold values imply statistical significance (p < 0.05)*.

There were a few rare instances where a patient with a platelet count ≥50,000/mm^3^ had an abnormal parameter on TEG corresponding to a hypocoagulable state (21.1% of those with platelet count of 50,000–99,999/mm^3^ and 8.3% of those with platelet count ≥100,000/mm^3^ were hypocoaguable on TEG, based on pathologist review). However, 7 of the 11 cases (63.6%) where a patient with a platelet count ≥50,000 had either prolonged R time, low alpha angle, or low MA were observed among patients with a diagnosis of acute leukemia or multiple myeloma.

No clinically significant procedure-related bleeding complications were identified (i.e., no BARC type 3, 4, or 5 bleeding events). Type 2 BARC bleeding events (3 femoral hematomas) were controlled with additional manual pressure to the arteriotomy site and use of non-invasive hemostatic devices (Neptune Pad®, Quick Clot®).

Only 3 patients underwent PCI and all 3 were in the mild thrombocytopenia group, with platelet counts of 59,000, 59,000, and 81,000/mm^3^, respectively. As no bleeding events occurred in this group, PCI was not associated with increased risk of bleeding.

Forty-two registered deaths (71.4%) occurred during the study period and were cancer-related (disease progression, sepsis), none were attributed to the cardiac catheterization or bleeding. The median OS was 12.6 months.

## Discussion

In our retrospective study of thrombocytopenic cancer patients who had undergone CA and concomitant TEG, platelet count <50,000/mm^3^ (compared to those with platelet count >100,000/mm^3^) was associated with worse OS at 24 months. However, hypocoagulability based on TEG was not associated with OS.

Despite including many patients with moderate or severe thrombocytopenia and with a perceived higher risk of bleeding, there were no substantial bleeding complications (BARC 3-5) among these 70 cancer patients undergoing CA. Thus, bleeding risk should not prevent interventional cardiologists from performing CA in cancer patients when clinically indicated.

Indications for performing TEG are not well-defined, with some operators using TEG as a cost-effective method to determine whether blood product administration before a procedure is indicated (22). One randomized controlled trial demonstrated that among cirrhotic patients with coagulopathy, TEG-guided blood product administration can decrease the frequency of administration of blood products without increasing the rate of bleeding complications (23).

As one might expect, hypocoagulability was noted more frequently among lower platelet groups in a step-wise fashion and a platelet count of <50,000/mm^3^ was associated with a hypocoagulable state on TEG, compared to a platelet count of ≥100,000/mm^3^ (see Table 3).

Similar hypocoagulability/abnormal TEG parameters among cancer patients with mild thrombocytopenia (i.e., platelet count 50–99,999/mm^3^) and those with platelet count >100,000/mm^3^ might suggest invasive testing [i.e., coronary angiography ± percutaneous coronary intervention (PCI)] in patients with mild thrombocytopenia is relatively safe and arguably obtaining a TEG when platelet count is >50,000/mm^3^ may be unnecessary. However, special consideration should be given to patients with acute leukemia or multiple myeloma that represent the rare instance where a patient with a platelet count ≥50,000/mm^3^ is hypocoagulable based on TEG. Further studies are required to determine if these patients may suffer from abnormal platelet function (as opposed to abnormal platelet count) that may predispose them to bleeding risk when undergoing CA.

**Figure 2:**
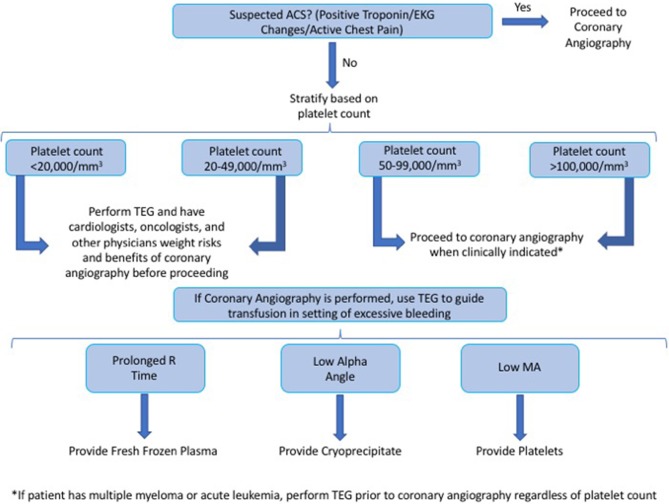
(Central illustration)—we propose the following algorithm, based on platelet count and TEG parameters, to guide the decision to proceed with CA among thrombocytopenic cancer patients and to guide the administration of blood products in the setting of bleeding complications.

In contrast to cardiothoracic or advanced endoscopic procedures where TEG is used to direct administration of blood products prior to the procedure, we also suggest the use of platelet count and TEG to risk stratify patients and obtain a more accurate risk/benefit assessment before proceeding with coronary angiography. As acute coronary syndrome (ACS) may be associated with a pro-thrombotic state (24) where pre-emptive platelet/FFP/cryoprecipitate administration may be harmful, in conjunction with the low risk of bleeding complications demonstrated in this study, we suggest only transfusing platelet/FFP/cryoprecipitate in the setting of bleeding among thrombocytopenic cancer patients undergoing CA. Figure 2 (Central Illustration) demonstrates our proposed algorithm, based on platelet count and TEG parameters, to guide the decision to proceed with CA among thrombocytopenic cancer patients and to guide administration of blood products in the setting of bleeding complications. We intend on performing future prospective studies to validate this algorithm.

We suggest obtaining a TEG in cancer patients with a platelet count of <50,000/mm^3^, or in those who carry the diagnosis of either acute leukemia or multiple myeloma regardless of platelet count.

A normal TEG might increase the confidence of the invasive cardiologist to not only perform CA and obtain additional physiological (iFR/FFR) and intraprocedural data (IVUS/OCT), but also to perform additional invasive procedures (such as endomyocardial biopsy for suspected myocarditis) if coronary anatomy does not suggest coronary lesions as the explanation for the positive cardiac biomarkers and/or clinical symptoms.

Notably, hypocoagulability on TEG was not associated with OS at 24 months, indicating that the results of TEG among cancer patients undergoing CA are not predictive of outcomes such as mortality and bleeding risk.

As opposed to hypocoagulability on TEG, platelet count was indeed associated with OS among cancer patients undergoing CA. A platelet count of <50,000/mm^3^ was a strong predictor of worse OS at 24 months compared with a platelet count of ≥100,000/mm^3^. This result is consistent with a previous study that demonstrated among cancer patients with cerebrovascular disease, thrombocytopenia at the time of the cerebrovascular disease diagnosis was associated with increased mortality (25). In light of our own findings, moderate-to-severe thrombocytopenia appears to be a poor prognostic indicator among cancer patients with vascular disease. Cancer patients suspected of having CAD with a platelet count of <50,000/mm^3^ may require assessment by multiple specialists (cardiologists, medical and surgical oncologists, internal medicine physicians, and critical care physicians) who can weigh the potential risks and benefits in the context of the overall survival before proceeding with the CA.

As survivorship appears to parallel the severity of thrombocytopenia, it appears that the highest risk of CA with the least benefit is among the most severely thrombocytopenic patients. CA should be reserved for those with a favorable cancer prognosis and where information obtained from CA can radically modify the medical management of the patient (i.e., selection of anticoagulation, anti-platelet therapy, chemotherapy, and/or radiation therapy) and thus impact OS.

## Limitations

In our retrospective study, not every single thrombocytopenic patient undergoing CA had a TEG prior to CA, introducing a selection bias (unstable patients or high-risk ACS patients underwent emergent CA without a concomitant TEG performed). TEG was performed in a limited fashion, only among those for whom coronary artery bypass graft (CABG) was not a viable alternative, and the interventional cardiologist had to be prepared for a high risk procedure (which may have theoretically been associated with an increased risk of bleeding). This led to a relatively small sample size, amongst whom we did not observe any bleeding complications.

Due to the complexity of the coagulation cascade, platelet function tests such as TEG may have limited utility. Furthermore, one study demonstrated that although the MA measurements are highly reproducible, measurements of R time and K are not as reproducible (26). Additionally, the TEG results of the same blood sample may vary from institution, likely owing to use of different hemostasis analyzers at different institutions.

Although the association with platelet count of <50,000/mm^3^ and overall survival may be confounded by cancer severity, it is important to note that prior history of congestive heart failure or cerebrovascular disease was associated with worse overall survival in this group, suggesting that cardiovascular/vascular disease may also significantly influence survivorship in this cohort may benefit from aggressive management of suspected cardiovascular disease.

Thirty-two of 72 patients (44.4%) were on aspirin prior to performing TEG, and 14 of 72 patients (19.44%) were on plavix prior to performing TEG, and this may have affected TEG results.

It is important to note that only 3 of 72 patients underwent PCI. However, in this unique cancer patient population, often times patients present with chest pain in unique scenarios (such as Takotsubo Cardiomyopathy) and thus do not require PCI.

## Conclusion

In conclusion, among cancer patients, a platelet count of 50,000–99,999/mm^3^ is not associated with a hypocoagulable state on TEG or a reduction in OS at 24 months when compared with >100,000/mm^3^, whereas a platelet count of <50,000/mm^3^ is associated with a decrease in OS at 24 months. While an abnormal TEG should not preclude any lifesaving procedure in cancer patient, it can help provide a more accurate risk/benefit assessment prior to CA. As survivorship appears to parallel the severity of thrombocytopenia, it appears that the most challenging balance risk/benefit before CA is in the severely thrombocytopenic patients.

Our study suggests that invasive cardiologists may be reassured when considering performing CA in cancer patients with a platelet count of ≥50,000/mm^3^, if the patient does not carry the diagnosis of acute leukemia or multiple myeloma (in which case, a TEG might be helpful regardless of the platelet count). Although no bleeding complications occurred among any of the patients in our sample, it may be wise to perform TEG among those who were most often noted to be hypocoaguable (i.e., those with a platelet count of <50,000/mm^3^ or acute leukemia or multiple myeloma), as these patients typically are not viable candidates for a surgical alternative such as coronary artery bypass graft (CABG), and if a bleeding complication is to occur during this procedure, then the interventional cardiologist must be fully prepared to address this with the prompt administration of the appropriate blood products based on TEG results.

TEG appears to have incremental value in severely thrombocytopenic patients and can help risk stratify these patients. Effective communication and teamwork between the oncologist, hemato/hematopathologist, invasive cardiologists, and other physicians is paramount to successful outcomes.

## Data Availability Statement

The datasets generated for this study are available on request to the corresponding author.

## Ethics Statement

The MD Anderson Institutional Review Board approved this study and waived the need for informed consent owing to its retrospective nature.

## Author Contributions

All authors listed have made a substantial, direct and intellectual contribution to the work, and approved it for publication.

### Conflict of Interest

The authors declare that the research was conducted in the absence of any commercial or financial relationships that could be construed as a potential conflict of interest.

## Abbreviations

CAD: Coronary Artery Disease
TEG: Thromboelastogram.
